# Deep learning approach for objective differentiation of kidney deficiency syndrome in reproductive age females: a tongue-face fusion model

**DOI:** 10.3389/fphys.2025.1701545

**Published:** 2025-11-25

**Authors:** Kaiwei Li, Zehong Qiu, Jialing Li, Feilin Deng, Kun Zou, Yihua Xu, Chen Huang, Ran Wang, Zhaoji Yu, Yuzhi Chen, Yingxuan Zhang, Zhuoliang Liu, Si Chen, Zhenning Su, Xiaojing Liu, Haiwang Wu, Xiaozhen Wu, Lilin Yang, Yanxi Huang, Songping Luo, Wu Zhou, Jie Gao

**Affiliations:** 1 The First Clinical Medical School of Guangzhou University of Chinese Medicine, Guangzhou, China; 2 Lingnan Medical Research Center, Guangzhou University of Chinese Medicine, Guangzhou, China; 3 School of Medical Information Engineering, Guangzhou University of Chinese Medicine, Guangzhou, China; 4 The First Affiliated Hospital of Guangzhou University of Chinese Medicine, Guangzhou, China; 5 Guangdong Clinical Research Academy of Chinese Medicine, Guangzhou, China; 6 State Key Laboratory of Traditional Chinese Medicine Syndrome, Guangzhou, China

**Keywords:** deep learning, syndrome differentiation, kidney deficiency syndrome, tongue-face fusion, gynecology of traditional Chinese medicine

## Abstract

**Background:**

Kidney deficiency syndrome (KDS) is the predominant syndrome associated with gynecological reproductive system diseases in traditional Chinese medicine (TCM). However, the diagnostic method is influenced by the subjective experience of doctors, which leads to the ambiguity in differentiation of KDS and poor effect for corresponding treatment.

**Objective:**

To explore an objective syndrome differentiation method for KDS in females of reproductive age through machine learning technique.

**Methods:**

We proposed a new deep learning method for the objective differentiation of KDS in females of reproductive age. First, we simultaneously acquired 376 pairs of tongue and facial images. We divided them into a Kidney deficiency syndrome (KDS, n = 182) group and a Non-Kidney deficiency syndrome (NKDS, n = 194) group. Then, we employed two parallel DenseNet structures to extract deep features from tongue and facial images. We further used a deep supervised network strategy to better stabilize the fusion of the two deep features. We used 5-fold cross-validation to evaluate the performance by six indicators: accuracy, precision, recall, F1 score, receiver operating characteristic (ROC) and area under the curve (AUC). Finally, external validation was conducted on an independent test set consisting of 130 patients with a 1:1 ratio of KDS to NKDS cases.

**Results:**

The model based on tongue images, facial images, and the tongue-face fusion model achieved AUCs of 71.45% ± 6.39%, 89.60% ± 3.33%, and 92.08% ± 4.51%, respectively, with the highest value observed in the fusion model. In external validation, the tongue-face fusion model attained an AUC of 83.53%.

**Conclusion:**

The deep learning network model with tongue-face fusion can effectively differentiate KDS.

## 1 Introduction

Fertility rates were projected to decline worldwide (Bhattacharjee et al., 2024). One reason is the increasing incidence of female reproductive disorders, including infertility (Santoro and Polotsky, 2025), pregnancy loss (Dimitriadis et al., 2020; Atik et al., 2023; Tong et al., 2024) and recurrent implantation failure (Ma et al., 2023). The safety and efficacy of traditional Chinese medicine (TCM) in the prevention and treatment of female reproductive disorders have been confirmed by clinical studies and basic experiments (Chen et al., 2022; Chen et al., 2024; Chen et al., 2024; Di et al., 2024; Tan et al., 2024). Despite the growing prevalence of Chinese patent medicines (CPMs) in clinical practice, a significant concern has arisen regarding their application. In certain cases, the syndrome-specific indications of CPMs are overlooked, with clinicians or patients predominantly focusing on disease-oriented applications rather than TCM syndrome differentiation. This practice potentially causes syndrome-medication mismatches that compromise therapeutic efficacy or induce adverse effects.

The term “syndrome” (*Zheng*) refers to a TCM-specific diagnostic concept that defines the disease location, nature, and stage of pathological progression. Clinicians determine a patient’s syndrome through a process called “syndrome differentiation” (*Bianzheng*), which involves inspection, auscultation and olfaction, inquiry, and palpation. Tailoring therapies according to the specific syndrome is the core principle of TCM, and has been demonstrated effective (Hu et al., 2022). Crucially, a single disease may manifest as multiple distinct syndromes, meaning different patients with the same disease diagnosis may present with divergent TCM syndrome classifications. These variations necessitate fundamentally different prescriptions, in some cases, even employing opposing therapeutic strategies. For a long time, the diagnostic methods of TCM have been difficult to popularize due to their strong subjectivity. Physicians and TCM students have been confronted with a variety of diagnostic teaching materials, making it challenging to form a unified and accurate understanding. Therefore, exploring the objective, accurate, and reliable development of syndrome differentiation methods is an urgent requirement for the development of TCM.

## 2 Related work

Artificial intelligence (AI) has emerged as a transformative tool in medical research and clinical practice (Qiu et al., 2025), offering potential solutions to the critical yet inherently subjective nature of syndrome differentiation in TCM. Recent advancements have specifically targeted the objective standardization of syndrome differentiation, employing machine learning to quantify traditionally experience-dependent diagnostic processes (Lu et al., 2024). A comprehensive review has enumerated fourteen machine learning methods and their uses in disease diagnosis and herb classification (Chen and He, 2022). Another study has highlighted the applications of computer vision and natural language processing (NLP) in TCM, with detailed explorations in tongue diagnosis, pulse diagnosis, and syndrome differentiation (Pan et al., 2024). Although standardized datasets for syndrome differentiation, such as TCMEval-SDT (Wang et al., 2025), TCMSSD (Huang et al., 2024) and TCM-SD (Mucheng Rena, 2022) are now available, methods that primarily depend on textual descriptions of symptoms remain subjective and struggle to achieve precise quantitative identification.

Inspection serves as the primary step in TCM diagnosis. Among its core components, tongue diagnosis has witnessed remarkable progress through AI approaches (Cheung Hui et al., 2007; Xu Jiatuo, 2024). By analyzing the characteristics of the tongue substance and coating, this method can reflect an individual’s health status and serves as a non-invasive diagnostic tool (Ma et al., 2019; Han et al., 2024; Wang et al., 2024; Liu et al., 2025; Sun et al., 2025; Yang et al., 2025). Recent studies have further combined the tongue images with oral microbiome to enhance diagnostic precision (Yuan et al., 2023; Dai et al., 2024; Deng et al., 2024; Deng et al., 2025; Zheng et al., 2025). Although two studies have shown that machine learning can effectively utilize tongue images to diagnose menstrual disorders (Liang et al., 2025) or predict the outcomes in frozen-thawed embryo transfer (Jinluan et al., 2024), the current research in gynecology is still insufficient.

The kidney deficiency syndrome (KDS) is the core syndrome of reproductive disorders in women (Zhu et al., 2014; Fan et al., 2023; Lai et al., 2023). Based on TCM theory and clinical practice, the inspection of tongue and face, which are considered external manifestations of internal health conditions, and reflect the body’s physiological and pathological states, is recognized as a key indicator for differentiating KDS in women. Specifically, the tongue and face provide distinct yet complementary information that can effectively reflect the presence of KDS. This theoretical and clinical foundation has motivated our methodological design, focusing on the integration of tongue and face features for syndrome differentiation.

Despite recent advancements in using AI for unimodal tongue (Xu Jiatuo, 2024) or facial analysis (Zhang et al., 2013; Zhao et al., 2014) in TCM, these single-modality approaches have limitations in achieving comprehensive diagnosis. To address this gap, we designed a novel deep learning framework that integrates tongue and facial images for KDS syndrome differentiation. We collected tongue and facial images of patients using instruments and determined the presence of KDS through the judgment of experienced clinical doctors. We then used the attention mechanism to extract dominant features from both types of data and perform deep feature fusion under a deeply supervised net. Our study leverages the strengths of both tongue and face inspection in TCM to develop an AI approach for KDS syndrome differentiation.

## 3 Methodology

### 3.1 Data collection

Following ethical approval (K-2023-082), we conducted prospective data collection at the gynecology clinic of the First Affiliated Hospital of Guangzhou University of Traditional Chinese Medicine between July and November 2023. Standardized tongue and facial images were captured using our independently developed imaging device, which had undergone and passed quality inspection by the Guangzhou Institute of Measurement and Testing Technology.

To obtain high-quality syndrome labels, the differentiation of TCM syndrome was performed by a nationally recognized TCM practitioner (certified by the Ministry of Human Resources and Social Security of the People’s Republic of China, the National Health Commission of the People’s Republic of China, and the National Administration of Traditional Chinese Medicine) during the diagnosis and treatment process. At the same time, the medical records of the participants were meticulously documented. During data organization, two frontline TCM clinical doctors were convened to review the syndrome judgment labels. In cases of disagreement, the medical records were re-evaluated to reach a consensus on syndrome determination.

The study enrolled female participants meeting the following criteria: Inclusion criteria: female patients of childbearing age, aged 18–49 years old. Exclusion criteria: (a) those who took food or medication before taking the photos that stained the tongue coating; (b) those who failed to expose most of the tongue surface due to tongue tension; (c) those who had poor image quality such as blurred focus, overexposure or underexposure; (d) those who had noticeably altered their skin tone through makeup. Although the same individual may exhibit different tongue or facial appearances at different stages, to avoid training errors, we retained only one set of tongue and facial images for each patient with a unique hospital ID number.

### 3.2 Label of the syndromes

Based on references including “*Clinic Terminology of Traditional Chinese Medical Diagnosis and Treatment—Part 2: Syndromes/Patterns (GB/T 16751.2-2021)*,” “*Basic Theory Nomenclature of Traditional Chinese Medicine (GB/T 20348-2006)*,” “*Clinical Terminology of Traditional Chinese Medicine—Part 6:Gynecology (GB/T 42467.6-2023)*,” we classified clinical experts’ syndrome differentiation results into two categories: KDS and non-KDS (NKDS). For example, the syndrome of dual deficiency of spleen and kidney is categorized as KDS, while syndrome of spleen deficiency as NKDS (Supplementary Table S1).

### 3.3 Image preprocessing

**FIGURE 1:**
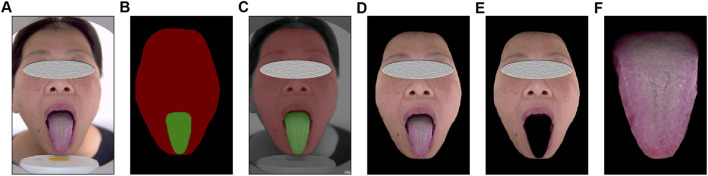
Image preprocessing. **(A)** Raw images included the patient’s face and tongue. **(B–D)** Regions of interest outlined using Labelme software. **(E,F)** Segmented images containing only the tongue of face respectively.

To facilitate the training of the deep learning network, we preprocessed the acquired tongue and facial images (Figure 1A). The tongue region and face region (Figures 1B–D) with corresponding pixel values outlined using Labelme software (http://labelme.csail.mit.edu/Release3.0/), were segmented from the original images to obtain images containing only the tongue or face (Figures 1E,F). To eliminate the effects caused by different feature scales and to make the model converge better, we normalized the data and resized the images to a uniform size of 224 × 224 pixels.

### 3.4 Proposed deep learning framework

**FIGURE 2:**
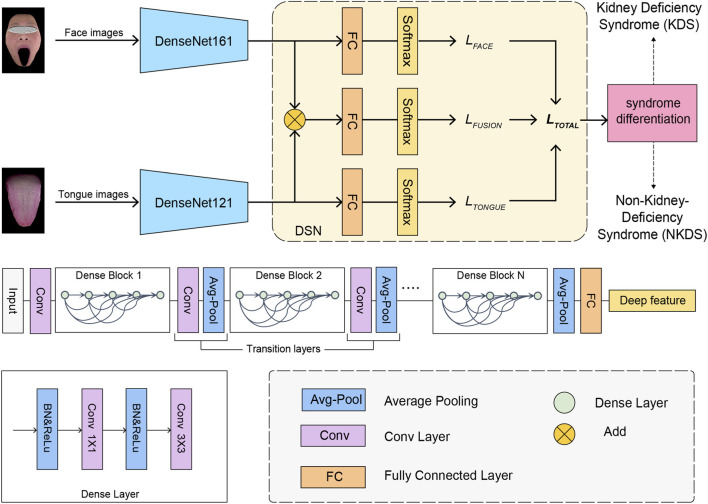
The deep learning framework for syndrome differentiation.

First, we screened various pretrained Convolutional Neural Networks (CNNs) architectures, such as ResNet, DenseNet, and EfficientNet to enhance the learning process (Szegedy et al., 2014; Szegedy et al., 2015; He et al., 2016; Huang et al., 2016; Howard et al., 2019; Dosovitskiy et al., 2020; Tan and Le, 2020; Liu et al., 2021; Liu et al., 2021; Tan and Le, 2021; Liu et al., 2022; Tu et al., 2022). Among all CNNs selected (Supplementary Table S2), DenseNet (Huang et al., 2016) demonstrated the best performance in single-modality test and was thus selected as the backbone model (Figure 2).

DenseNet consists of multiple Dense Blocks and Transition Layers between these blocks. Within each Dense Block, the output feature maps of each layer are directly passed to all subsequent layers, forming dense feature connections. This design allows the network to utilize features more effectively and avoid feature loss and repeated calculations. The densely connected approach, where any two layers are directly connected, helps alleviate the vanishing-gradient problem, enhances feature propagation and reuse, and reduces network parameters. Consequently, we employed two parallel DenseNet structures to extract deep features from tongue and facial images.

### 3.5 Fusion strategy

To integrate the information from the tongue and face to enhance the differentiation performance, we employed a Deep Supervision Network (DSN) (Liu et al., 2022) for the design of modality fusion (Figure 2). The DSN was originally proposed to provide comprehensive direct supervision of hidden layers, aiming to enhance the learning process of CNNs by increasing the directness and transparency of hidden layers. Inspired by the deep supervision in DSN, we can directly supervise deep features from multi-modal images, offering strong regularization for the deep features of tongue and facial images to improve final classification. Our DSN fusion framework uses a cross-entropy loss function $L_{\text{cross}-\text{entropy}}$ for the concatenated deep features from tongue and facial images for classification. The losses for the tongue and face streams can be seen as separate loss functions in the network that require supervision.

$$L_{\text{cross}-\text{entropy}}=-\sum_{i=1}^{N}y_{i}\cdot \log\left({\hat{y}}_{i}\right)$$ (1)

The weighted cross-entropy loss, as shown in Equation 1, is computed between the true label $y_{i}$ , and predicted label ${\hat{y}}_{i}$ . Thus, the total loss function $L_{TOTAL}$ of our framework is defined as follows. Here, $L_{TONGUE}$ and $L_{FACE}$ are the cross-entropy loss functions for the concatenated deep features of tongue and facial images, respectively. $L_{FUSION}$ represents the concatenation of both.

$$L_{TOTAL}=L_{FUSION}+L_{FACE}+L_{TONGUE}$$

### 3.6 Implementation and training strategy

The proposed network structure was implemented using the platform PyTorch (version 2.2.1) in a programming environment on the server graphics card with NVIDIA GeForce RTX 3090 (24 GB) GPU. The model was trained for 100 epochs with a batch size of 16 and an initial learning rate of 2e-4. We used the AdamW optimiser with L2 regularization set to 1e-4, which is widely used in training deep learning models due to its stability and fast convergence. The cosine annealing method was used to decay the learning rate to a minimum of 1e-6. The model weights were initialized using a backbone network pre-trained on ImageNet (Russakovsky et al., 2014).

### 3.7 Evaluation metric and visualization

Performance evaluation was carried out using a 5-fold cross-validation method and determined by calculating the mean and variance of each evaluation index, evaluated by the following six indicators: (a)Accuracy; (b) Precision; (c) Recall; (d) F1 score; (e) Receiver Operating Characteristic (ROC); (f) Area under Curve (AUC).

$$\text{Accuracy}=\frac{\text{TP}+\text{TN}}{\text{TP}+\text{TN}+\text{FP}+\text{FN}}$$ (a)

$$\text{Precision}=\frac{\text{TP}}{\text{TP}+\text{FP}}$$ (b)

$$\text{Recall}=\frac{\text{TP}}{\text{TP}+\text{FN}}$$ (c)

$$F1\text{ score}=\frac{\text{Precision}\times \text{Recall}}{\text{Precision}+\text{Recall}}$$ (d)

TP, FP, TN, and FN represent True Positive, False Positive, True Negative, and False Negative, respectively. Accuracy is the proportion of correctly classified positive and negative cases among all samples. Precision refers to the proportion of correctly classified positive cases among all positive cases predicted by the model. Recall is the proportion of positive cases correctly predicted by the model among all positive samples. The F1 score is a balanced metric for assessing the accuracy of a classification model, considering both Precision and Recall, and thus can be viewed as the harmonic mean of the two. The ROC curve combines sensitivity and specificity, visually reflecting the relationship between them in a curve form, providing a visual representation that facilitates the analysis of model performance. The AUC value is defined as the area under the ROC curve, and the larger its value, the better the model’s classification effectiveness.

## 4 Experiments

**FIGURE 3:**
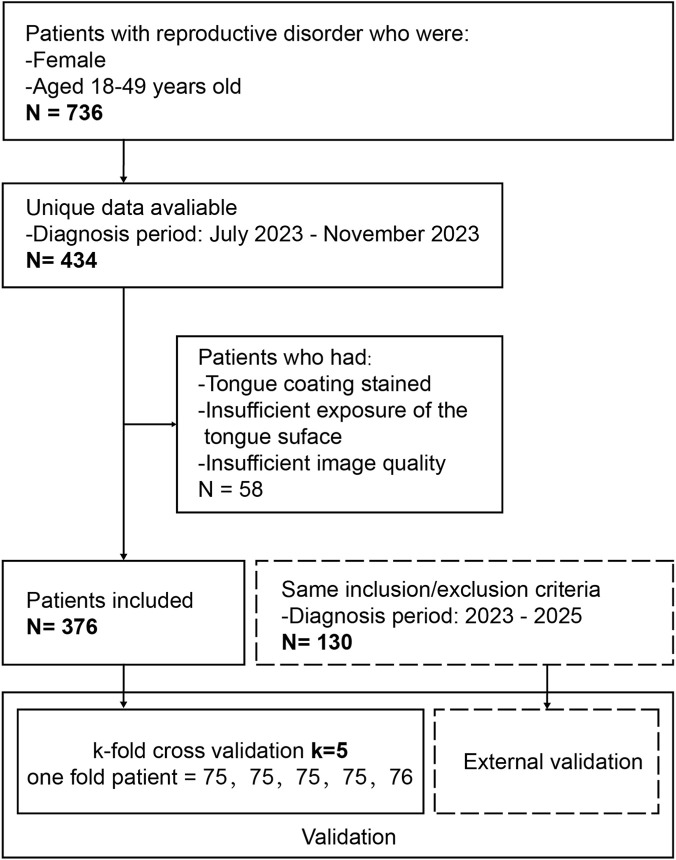
Flowcharts show patient inclusion.

The study included a total of 376 cases, which were divided into KDS (n = 182) and NKDS (n = 194) groups. The mean age ± standard deviation for the KDS and NKDS groups was 35.09 ± 5.43 and 33.37 ± 5.68, respectively. The mean age of the KDS group was slightly higher than that of the NKDS group (*P* < 0.05, t-test). This finding may be attributed to the fact that, in TCM theory, kidney essence gradually declines with age, which can lead to the development of KDS. We divided the 376 patients with tongue and facial images into a training set and a validation set, using 5-fold cross-validation for performance assessment (Figure 3).

**FIGURE 4:**
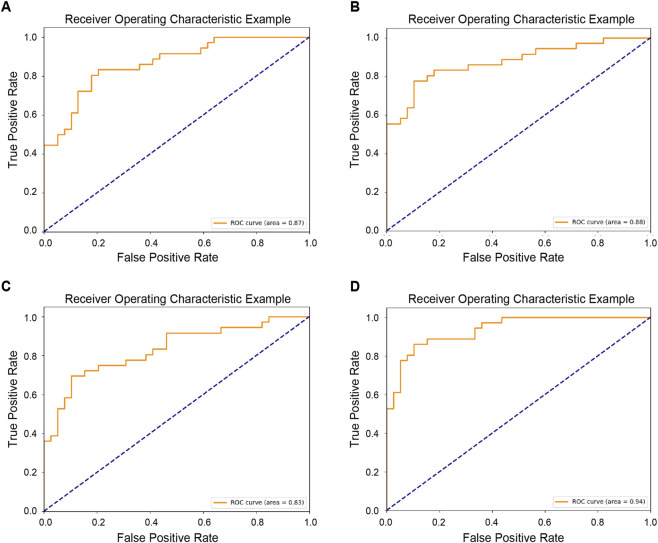
ROC of four models using both tongue and facial images. **(A)** Model concat_121_161. **(B)** Model add_121_161. **(C)** Model DSNconcat_121_161. **(D)** Model DSNadd_121_161.

**FIGURE 5:**
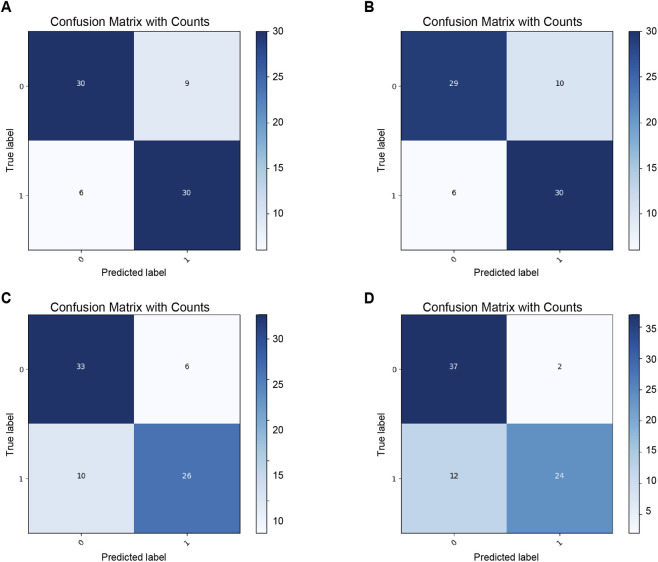
Confusion matrices of four models using both tongue and facial images. **(A)** Model concat_121_161. **(B)** Model add_121_161. **(C)** Model DSNconcat_121_161. **(D)** Model DSNadd_121_161.

For comparison, we also evaluated the classification performance using single-modality (tongue or face) data. After balancing performance against parameter count, we selected DenseNet-121 and DenseNet-161 as the optimal dual branch configuration for deep feature extraction (Table 1). Subsequently, we compared concatenation-based, addition-based (Ramachandram and Taylor, 2017), and DSN-based fusion strategies (Table 2; Figures 4, 5).

**TABLE 1:** Performance of models using single-modality data.

Model name	Accuracy (%)	Precision (%)	Recall (%)	F1 score (%)	AUC (%)
face_DenseNet121	81.39 ± 6.15	82.57 ± 4.62	78.08 ± 11.09	80.00 ± 7.14	88.29 ± 4.66
face_DenseNet169	80.57 ± 6.40	82.23 ± 8.21	76.91 ± 7.03	79.34 ± 6.69	88.36 ± 4.64
face_DenseNet161	79.52 ± 5.65	79.88 ± 6.47	77.45 ± 8.69	78.44 ± 6.45	88.51 ± 4.90
face_DenseNet201	81.66 ± 3.85	81.77 ± 4.27	80.26 ± 7.56	80.81 ± 4.28	89.60 ± 3.33
tongue_DenseNet161	61.18 ± 4.84	60.80 ± 6.21	57.79 ± 10.86	58.69 ± 6.80	68.29 ± 4.49
tongue_DenseNet169	63.05 ± 7.47	63.23 ± 8.30	56.59 ± 12.17	59.33 ± 9.70	68.55 ± 4.66
tongue_DenseNet201	64.38 ± 5.99	65.56 ± 5.65	54.43 ± 13.61	58.98 ± 10.25	70.43 ± 4.93
tongue_DenseNet121	65.44 ± 6.07	65.00 ± 4.55	61.55 ± 17.44	62.26 ± 10.71	71.45 ± 6.39

#The values reported in tables represent mean values ± standard deviation.

**TABLE 2:** Performance of models using both tongue and facial images.

Model name	Accuracy (%)	Precision (%)	Recall (%)	F1 score (%)	AUC (%)
concat_121_161	81.39 ± 5.32	79.96 ± 3.72	81.87 ± 9.09	80.81 ± 6.14	90.38 ± 3.93
add_121_161	84.05 ± 6.23	85.41 ± 8.05	81.34 ± 7.03	83.16 ± 6.50	90.94 ± 4.55
DSNconcat_121_161	74.75 ± 3.51	89.88 ± 7.76	55.06 ± 11.49	67.33 ± 7.01	87.80 ± 5.45
DSNadd_121_161	81.66 ± 6.84	92.76 ± 4.52	67.09 ± 1.12	77.66 ± 8.84	92.08 ± 4.51

#The values reported in tables represent mean values ± standard deviation.

Under single-modality conditions, facial image classification consistently outperforms tongue image classification, likely due to the higher information density of facial images compared to tongue images. After deep-feature fusion, the combined representation of tongue and facial images achieves a further performance improvement. Notably, the introduction of the DSN imposes direct supervision on the deep features of both modalities, enhancing their robustness and, consequently, elevating the overall classification accuracy.

We further conducted external validation on a test set comprising 130 patients with a 1:1 positive-to-negative ratio (Table 3). The results showed that although the AUC of the tongue-face fusion model decreased, it remained above 80%, confirming a certain level of model stability.

**TABLE 3:** External validation of models using both tongue and facial images.

Model name	Accuracy (%)	Precision (%)	Recall (%)	F1 score (%)	AUC (%)
DSNconcat_121_161	68.46	71.43	61.54	66.12	79.43
DSNadd_121_161	75.38	72.60	81.54	76.81	83.53

## 5 Discussion

In this study, we proposed a new deep learning method for the objective differentiation of KDS in females of reproductive ages, aiming to improve the efficacy of TCM treatment. Compared to models that rely solely on tongue or facial information, we found that the model integrating both tongue and facial images demonstrates better efficacy in differentiating KDS. Our results are consistent with TCM theory and clinical practice, confirming that the inspection of tongue and face is a key indicator for differentiating KDS in women.

Inspection is the foremost among the four diagnostic methods in TCM and imposes minimal burden on the patient. The examination of the face and tongue is an important part of inspection in TCM and contributes greatly to syndrome differentiation. The exterior of the human body and the *zang*(脏)-*fu*(腑) viscera are connected by meridians. Any disharmony between qi and blood, yin and yang, zang and fu will be reflected on the outside of the body. Observation of the tongue and face can reveal the deficiencies and excesses of the viscera, the smoothness or obstruction of the meridians, the abundance or deficiency of qi and blood, and the sufficiency or deficiency of body fluids.

Our CNN-based diagnostic models using tongue or facial images have shown promising performance (the highest AUC for each >0.70), suggesting that kidney deficiency syndrome in TCM gynecology can be differentiated to some extent by analyzing the tongue or face alone. Unexpectedly, the performance of the facial-modality model (using DenseNet121, AUC = 89.60% ± 3.33%) was notably higher than the tongue-modality one (using DenseNet121, AUC = 71.45% ± 6.39%). This discrepancy can be attributed to several factors. First, the image captured by the device is a two-dimensional view. Moreover, the spatial relationship between the image capture device and the patient’s extended tongue is relatively fixed. As a result, the machine cannot change the angle for tongue observation as flexibly as a clinician can. This inflexibility means that the actual spatial information of the acquired image is compressed, and the machine learns limited information about the tongue. In contrast, when recognizing facial images, the model focuses on a much wider range of regions.

TCM emphasizes the integration of the four examinations, including local examinations of the head, whole face, eyes, ears, mouth, nose, tongue, neck, body, limbs and skin. Previous research has mostly analyzed tongue and face information independently and has mostly been applied to disease diagnosis rather than syndrome differentiation. Therefore, our study used a DSN to fuse tongue and face features to construct a gynecological kidney deficiency syndrome differentiation model for women of reproductive age. The results show that the model using integrated information has higher performance than others, suggesting that a comprehensive analysis of both tongue and face information is more conducive to syndrome differentiation. In external validation, the tongue-face fusion model maintained its efficacy, confirming a certain level of model stability.

This study has some limitations. First, this study included a single-center sample, with a relatively small dataset. Due to the limited availability of consultation slots with the nationally recognized TCM practitioner, the initial sample size was constrained. Furthermore, after implementing a rigorous image screening process, the number of samples ultimately included in model construction was relatively small. Considering that a small sample size could lead to overfitting, we additionally utilized a set of images—also sourced from the clinical data of the nationally recognized TCM expert but previously unseen by the model—for external validation. Meanwhile, to enhance the generalizability of our research findings, we have initiated multi-center sample collection and have already commenced work at Sun Yat-sen Memorial Hospital of Sun Yat-sen University in Guangzhou and Shenzhen Maternity & Child Healthcare Hospital in China. During this multi-center data collection process, we adopted a TCM syndrome information scale, validated through expert consensus at the national level, to standardize syndrome differentiation across different centers. Second, due to the limited sample size, we focused only on the KDS, which is the most common TCM syndrome of females productive disorders. We were also not able to further subclassify KDS into subtypes, such as kidney-yin deficiency syndrome and kidney-yang deficiency syndrome, each of which has distinct clinical manifestations. For instance, kidney-yang deficiency syndrome typically presents with a pale tongue and a white coating, while kidney-yin deficiency syndrome is characterized by a red tongue with a thin coating. As our dataset continues to expand, we plan to further identify and distinguish these syndromes, using fine-grained recognition methods. Third, women in different physiological periods exhibit unique syndrome characteristics. There are subtle differences in KDS presentation during reproductive years versus menopause, as well as menstrual and pregnancy periods. Fourth, we did not incorporate the morphological features of the tongue, such as tongue coating and tongue color. Integrating these features may further improve the performance of our model. Fifth, our current approach relies solely on inspection methods. We have not yet incorporated patients’ medical records or examination results. Future research will consider integrating multiple cross-modal information sources to further enhance the performance of syndrome differentiation.

## 6 Conclusion

In conclusion, our study simulated the clinical process of TCM by designing an integration of tongue-facial features, and validated its effectiveness in enhancing performance compared to single-modality approaches through a data-driven method. Our results show the potential of deep learning models to differentiate KDS based on objective tongue and facial images.

## Data Availability

The raw data supporting the conclusions of this article will be made available by the authors, without undue reservation.

## References

[B1] Atik R. B. Christiansen O. B. Elson J. Kolte A. M. Lewis S. Middeldorp S. (2023). Eshre guideline: recurrent pregnancy loss: an update in 2022. Hum. Reprod. Open 2023, 1–7. 10.1093/hropen/hoad002 PMC9982362 36873081

[B2] Bhattacharjee N. V. Schumacher A. E. Aali A. Abate Y. H. Abbasgholizadeh R. Abbasian M. (2024). Global fertility in 204 countries and territories, 1950–2021, with forecasts to 2100: a comprehensive demographic analysis for the global burden of disease study 2021. Lancet 403, 2057–2099. 10.1016/S0140-6736(24)00550-6 38521087 PMC11122687

[B3] Chen H. He Y. (2022). Machine learning approaches in traditional Chinese medicine: a systematic review. Am. J. Chin. Med. 50, 91–131. 10.1142/S0192415X22500045 34931589

[B4] Chen X. Hao C. Deng W. Bai H. Li Y. Wang Z. (2022). Effects of the Zishen Yutai Pill compared with placebo on live births among women in a fresh embryo transfer cycle: a randomized controlled trial. Obstetrics Gynecol*.* 139, 192–201. 10.1097/AOG.0000000000004658 34991130 PMC8759541

[B5] Chen S. Xue X. Zhang H. Huang X. Lin X. He J. (2024). Jianwei shoutai pills alleviates miscarriage by modulating gut microbial production of bas and nlrp3-inflammasome at the maternal-fetal interface of rats. Phytomedicine 135, 156000. 10.1016/j.phymed.2024.156000 39293366

[B6] Chen X. Shi Y. Li H. Gong F. Yao C. Bai H. (2024). Effects of the Zishen Yutai Pill on live births compared with placebo among infertile women with frozen-thawed embryo transfer cycle: a multicentre double-blind randomized controlled trial. Phytomedicine 135, 156072. 10.1016/j.phymed.2024.156072 39348779

[B7] Cheung Hui S. He Y. Doan Thi Cam Thach (2007). “Machine learning for tongue diagnosis, 1–5. 10.1109/ICICS.2007.4449631

[B8] Dai S. Guo X. Liu S. Tu L. Hu X. Cui J. (2024). Application of intelligent tongue image analysis in conjunction with microbiomes in the diagnosis of mafld. Heliyon 10, e29269. 10.1016/j.heliyon.2024.e29269 38617943 PMC11015139

[B9] Deng J. Dai S. Liu S. Tu L. Cui J. Hu X. (2024). Application of tongue image characteristics and oral-gut microbiota in predicting pre-diabetes and type 2 diabetes with machine learning. Front. Cell. Infect. Microbiol. 14, 1477638–14. 10.3389/fcimb.2024.1477638 39559704 PMC11570591

[B10] Deng J. Dai S. Liu S. Tu L. Cui J. Hu X. (2025). Clinical study of intelligent tongue diagnosis and oral microbiome for classifying tcm syndromes in masld. Chin. Med. 20, 78. 10.1186/s13020-025-01118-w 40442807 PMC12121174

[B11] Di X. Duan Z. Ma Y. Song X. Hao Y. Li G. (2024). Jiawei shoutai pill promotes decidualization by regulating the sgk1/enac pathway in recurrent spontaneous abortion. J. Ethnopharmacol. 318, 116939. 10.1016/j.jep.2023.116939 37479068

[B12] Dimitriadis E. Menkhorst E. Saito S. Kutteh W. H. Brosens J. J. (2020). Recurrent pregnancy loss. Nat. Rev. Dis. Prim. 6, 98. 10.1038/s41572-020-00228-z 33303732

[B13] Dosovitskiy A. Beyer L. Kolesnikov A. Weissenborn D. Zhai X. Unterthiner T. (2020). An image is worth 16x16 words: transformers for image recognition at scale arXiv:2010.11929. 10.48550/arXiv.2010.11929

[B14] Fan H. Y. Chen S. S. Li X. Q. Huang L. Y. (2023). Efficacy of traditional Chinese herbs combined with bioelectrical stimulation on patients with kidney deficiency and blood stasis type thin endometrium: a retrospective observational study. Eur. Rev. Med. Pharmacol. Sci. 27 (7), 2971–2979. 10.26355/eurrev_202304_31929 37070898

[B15] Han B. Chang Y. Tan R. Han C. (2024). Evaluating deep learning techniques for identifying tongue features in subthreshold depression: a prospective observational study. Front. Psychiatry 15, 1361177–15. 10.3389/fpsyt.2024.1361177 39176227 PMC11338782

[B16] He K. Zhang X. Ren S. Sun J. (2016). “Deep residual learning for image recognition. 770–778. 10.1109/cvpr.2016.90

[B17] Howard A. Sandler M. Chu G. Chen L. Chen B. Tan M. (2019). Searching for mobilenetv3 arXiv:1905.02244v5. 10.48550/arXiv.1905.02244

[B18] Hu Y. Gu S. Yuan X. Li H. Yuan C. Ye Q. (2022). Traditional Chinese medicine syndrome differentiation and treatment by stages of parkinson’s disease: study protocol for a multicentre, randomized, double-blind, placebo-controlled clinical trial. Chin. Med. 17, 68. 10.1186/s13020-022-00625-4 35698234 PMC9190110

[B19] Huang G. Liu Z. van der Maaten L. Weinberger K. Q. (2016). Densely connected convolutional networks arXiv:1608.06993. 10.48550/arXiv.1608.06993

[B20] Huang L. Wang Q. Duan Q. Shi W. Li D. Chen W. (2024). Tcmssd: a comprehensive database focused on syndrome standardization. Phytomedicine 128, 155486. 10.1016/j.phymed.2024.155486 38471316

[B21] Jinluan W. A. N. G. Zhiling G. U. O. Qinhua Z. H. A. N. Hua Y. A. N. Liping T. U. Jiatuo X. U. (2024). Correlation between tongue and pulse indicators and the outcome of live birth in frozen-thawed embryo transfer. Digit. Chin. Med. 7, 68–78. 10.1016/j.dcmed.2024.04.008

[B22] Lai Y. Zhang Y. Zhang H. Chen Z. Zeng L. Deng G. (2023). Modified shoutai pill inhibited ferroptosis to alleviate recurrent pregnancy loss. J. Ethnopharmacol. 319, 117028. 10.1016/j.jep.2023.117028 37597678

[B23] Liang A. Ge J. Liu Z. Han X. Hou S. Li G. (2025). Reliability of noninvasive hyperspectral tongue diagnosis for menstrual diseases using machine learning method. Sci. Rep. 15, 6138. 10.1038/s41598-025-90679-9 39979510 PMC11842557

[B24] Liu Z. Hu H. Lin Y. Yao Z. Xie Z. Wei Y. (2021). Swin transformer v2: scaling up capacity and resolution arXiv:2111.09883. 10.48550/arXiv.2111.09883

[B25] Liu Z. Lin Y. Cao Y. Hu H. Wei Y. Zhang Z. (2021). Swin transformer: hierarchical vision transformer using shifted windows arXiv:2103.14030. 10.48550/arXiv.2103.14030

[B26] Liu Z. Mao H. Wu C. Feichtenhofer C. Darrell T. Xie S. (2022). A convnet for the 2020s arXiv:2201.03545. 10.48550/arXiv.2201.03545

[B27] Liu Y. Fan L. Zhao M. Wei D. Zhao M. Dong Y. (2025). Study on a traditional Chinese medicine constitution recognition model using tongue image characteristics and deep learning: a prospective dual-center investigation. Chin. Med. 20, 84. 10.1186/s13020-025-01126-w 40506765 PMC12160370

[B28] Lu L. Lu T. Tian C. Zhang X. (2024). Ai: bridging ancient wisdom and modern innovation in traditional Chinese medicine. JMIR Med. Inf. 12, e58491. 10.2196/58491 38941141 PMC11245652

[B29] Ma J. Wen G. Wang C. Jiang L. (2019). Complexity perception classification method for tongue constitution recognition. Artif. Intell. Med. 96, 123–133. 10.1016/j.artmed.2019.03.008 31164206

[B30] Ma J. Gao W. Li D. (2023). Recurrent implantation failure: a comprehensive summary from etiology to treatment. Front. Endocrinol. 13, 1061766–21. 10.3389/fendo.2022.1061766 36686483 PMC9849692

[B31] Mucheng Rena H. H. Y. Z. (2022). Tcm-sd: a large dataset for syndrome diferentiation in traditional Chinese medicine. CoRR, 2–18. 10.48550/arXiv.2203.10839

[B32] Pan D. Guo Y. Fan Y. Wan H. (2024). Development and application of traditional Chinese medicine using ai machine learning and deep learning strategies. Am. J. Chin. Med. 52, 605–623. 10.1142/S0192415X24500265 38715181

[B33] Qiu Y. Ma W. Wang H. Tham Y. Wong T. Y. (2025). The landscape of medical ai in China. NEJM AI. 10.1056/AIp2401234

[B34] Ramachandram D. Taylor G. W. (2017). Deep multimodal learning: a survey on recent advances and trends. IEEE Signal Process. Mag. 34, 96–108. 10.1109/MSP.2017.2738401

[B35] Russakovsky O. Deng J. Su H. Krause J. Satheesh S. Ma S. (2014). Imagenet large scale visual recognition challenge arXiv:1409.0575. 10.48550/arXiv.1409.0575

[B36] Santoro N. Polotsky A. J. (2025). Infertility evaluation and treatment. N. Engl. J. Med. 392, 1111–1119. 10.1056/NEJMcp2311150 40073310

[B37] Sun X. Huang L. Qu L. Chen C. Zeng X. Zhou Z. (2025). Development of a tongue image-based machine learning tool for the diagnosis of colorectal cancer: a prospective multicentre clinical cohort study. IEEE J. Biomed. Health Inf. 1–15. 10.1109/JBHI.2025.3585552 40601462

[B38] Szegedy C. Liu W. Jia Y. Sermanet P. Reed S. Anguelov D. (2014). Going deeper with convolutions arXiv:1409.4842.

[B39] Szegedy C. Vanhoucke V. Ioffe S. Shlens J. Wojna Z. (2015). Rethinking the inception architecture for computer vision arXiv:1512.00567v3.

[B40] Tan M. Le Q. V. (2020). Effcientnet: rethinking model scaling for convolutional neural networks arXiv:1905.11946.

[B41] Tan M. Le Q. V. (2021). Effcientnetv2: smaller models and faster training arXiv:2104.00298.

[B42] Tan J. Xiong Y. Zhao P. Liu C. Ren Y. Chen M. (2024). Safety of herbal medicines used in early gestations among the Chinese population: a population-based cohort study. Phytomedicine 135, 156197. 10.1016/j.phymed.2024.156197 39515097

[B43] Tong F. Wang Y. Gao Q. Zhao Y. Zhang X. Li B. (2024). The epidemiology of pregnancy loss: global burden, variable risk factors, and predictions. Hum. Reprod. 39, 834–848. 10.1093/humrep/deae008 38308812

[B44] Tu Z. Talebi H. Zhang H. Yang F. Milanfar P. Bovik A. (2022). Maxvit: multi-axis vision transformer arXiv:2204.01697v4. 10.48550/arXiv.2204.01697

[B45] Wang L. Zhang Q. Zhang P. Wu B. Chen J. Gong J. (2024). Development of an artificial intelligent model for pre-endoscopic screening of precancerous lesions in gastric cancer. Chin. Med. 19, 90. 10.1186/s13020-024-00963-5 38951913 PMC11218324

[B46] Wang Z. Hao M. Peng S. Huang Y. Lu Y. Yao K. (2025). Tcmeval-sdt: a benchmark dataset for syndrome differentiation thought of traditional Chinese medicine. Sci. Data 12, 437. 10.1038/s41597-025-04772-9 40082443 PMC11906624

[B47] Xu Jiatuo J. T. L. S. Tao J. Shi L. (2024). Research status and prospect of tongue image diagnosis analysis based on machinelearning. Digit. Chin. Med. 7 (01), 3–12. 10.1016/j.dcmed.2024.04.002

[B48] Yang L. Dong Q. Lin D. Lü X. (2025). Tonguenet: a multi-modal fusion and multi-label classification model for traditional Chinese medicine tongue diagnosis. Front. Physiol. 16, 1527751–15. 10.3389/fphys.2025.1527751 40352152 PMC12061702

[B49] Yuan L. Yang L. Zhang S. Xu Z. Qin J. Shi Y. (2023). Development of a tongue image-based machine learning tool for the diagnosis of gastric cancer: a prospective multicentre clinical cohort study. EClinicalMedicine 57, 101834. 10.1016/j.eclinm.2023.101834 36825238 PMC9941057

[B50] Zhang B. Wang X. Karray F. Yang Z. Zhang D. (2013). Computerized facial diagnosis using both color and texture features. Inf. Sci. 221, 49–59. 10.1016/j.ins.2012.09.011

[B51] Zhao C. Li G. Li F. Wang Z. Liu C. (2014). Qualitative and quantitative analysis for facial complexion in traditional Chinese medicine. Biomed. Res. Int. 2014, 207589–17. 10.1155/2014/207589 24967342 PMC4054802

[B52] Zheng W. Yinhang W. Jian C. Congjian J. Zhanbo Q. Nianga J. (2025). Characteristics of tongue images and tongue coating bacteria in patients with colorectal cancer. BMC Microbiol. 25, 285. 10.1186/s12866-025-04014-3 40350439 PMC12066039

[B53] Zhu X. Zhao L. Fu J. Su M. (2014). “nourishing the kidney” and the treatment of recurrent pregnancy loss using traditional Chinese medicine. Int. J. Gynaecol. Obstet. 127, 90–91. 10.1016/j.ijgo.2014.06.002 24994494

